# Chemotropism Assays for Plant Symbiosis and Mycoparasitism Related Compound Screening in *Trichoderma atroviride*

**DOI:** 10.3389/fmicb.2020.601251

**Published:** 2020-11-27

**Authors:** Dubraska Moreno-Ruiz, Alexander Lichius, David Turrà, Antonio Di Pietro, Susanne Zeilinger

**Affiliations:** ^1^Department of Microbiology, University of Innsbruck, Innsbruck, Austria; ^2^Departamento de Genética, Universidad de Córdoba, Córdoba, Spain

**Keywords:** secondary metabolites, fungal plant pathogens, mycoparasitism, *Trichoderma atroviride*, chemotropism, plant symbiosis, host sensing

## Abstract

*Trichoderma atroviride* is a mycoparasitic fungus used as biological control agent to protect plants against fungal pathogens. Successful biocontrol is based on the perception of signals derived from both the plant symbiont and the fungal prey. Here, we applied three different chemotropic assays to study the chemosensing capacity of *T. atroviride* toward compounds known or suspected to play a role in the mycoparasite/plant or host/prey fungal interactions and to cover the complete spectrum of *T. atroviride* developmental stages. Purified compounds, including nutrients, the fungal secondary metabolite 6-amyl-α-pyrone (6-pentyl-α-pyrone, 6-PP) and the plant oxylipin 13-(s)-HODE, as well as culture supernatants derived from fungal preys, including *Rhizoctonia solani*, *Botrytis cinerea* and *Fusarium oxysporum*, were used to evaluate chemotropic responses of conidial germlings, microcolonies and fully differentiated mycelia. Our results show that germlings respond preferentially to compounds secreted by plant roots and *T. atroviride* itself than to compounds secreted by prey fungi. With the progression of colony development, host plant cues and self-generated signaling compounds remained the strongest chemoattractants. Nevertheless, mature hyphae responded differentially to certain prey-derived signals. Depending on the fungal prey species, chemotropic responses resulted in either increased or decreased directional colony extension and hyphal density at the colony periphery closest to the test compound source. Together these findings suggest that chemotropic sensing during germling development is focused on plant association and colony network formation, while fungal prey recognition develops later in mature hyphae of fully differentiated mycelium. Furthermore, the morphological alterations of *T. atroviride* in response to plant host and fungal prey compounds suggest the presence of both positive and negative chemotropism. The presented assays will be useful for screening of candidate compounds, and for evaluating their impact on the developmental spectrum of *T. atroviride* and other related species alike. Conidial germlings proved particularly useful for simple and rapid compound screening, whereas more elaborate microscopic analysis of microcolonies and fully differentiated mycelia was essential to understand process-specific responses, such as plant symbiosis and biocontrol.

## Introduction

*Trichoderma atroviride* employs several mechanisms to counteract soil-dwelling plant pathogenic fungi, including the competition for space and resources, rhizosphere modification, secretion of antifungal compounds, and direct mycoparasitism (Benítez et al., 2004). Biocontrol by competition for resources describes the more effective mobilization and absorption of nutrients from the soil by *Trichoderma* than by fungal plant pathogens inhabiting the same space (Chet and Inbar, 1994). In addition, a higher resistance against fungistatic secondary metabolites secreted by other fungal species, plants or soil bacteria provides *Trichoderma* with a fitness advantage against other fungal species (Benítez et al., 2004). Active rhizosphere modification by *Trichoderma* through the secretion of toxic secondary metabolites and compounds with fungistatic or antifungal properties inhibits the growth of other fungi and other microorganisms with less effective ABC pumps or enzyme secretion systems (Delgado-Jarana et al., 2000, 2002; Benítez et al., 2004). Numerous *Trichoderma* species employ volatile and non-volatile compounds, hydrolytic enzymes, and peptaibols for this purpose (Monte, 2001; Wiest et al., 2002; Howell, 2003).

Mycoparasitism describes the direct attack of one fungus (the prey) by another fungus (the mycoparasite) with the aim to exploit the prey as a nutrient source. Key stages of the process include prey recognition, positive chemotropic growth toward it, chemical and physical attack – the combination of both usually results in the killing of fungal prey hyphae – and finally nutrient uptake. The pre-contact chemical attack includes the induced production and mass release of cell wall degrading enzymes (Viterbo et al., 2002; Howell, 2003; Harman et al., 2004), whereas physical attack during the contact phase is accompanied by significant morphological changes that may include intense branching, hyphal coiling or the formation of appressorium-like structures (Lu et al., 2004).

Although positive chemotropism in response to prey-derived stimuli has been recognized as a key initiating feature of mycoparasitism early on (Chet et al., 1981), the cellular and molecular details of prey sensing are still poorly understood. Inter-species recognition in fungi has been linked to remote sensing of cell wall components (Chet et al., 1981; Zeilinger et al., 1999; Viterbo et al., 2002; Harman et al., 2004; Vinale et al., 2008). It is accepted that fungi permanently secrete low amounts of extracellular chitinases that release cell wall oligomers from target fungi in the vicinity. These, in turn, are thought to serve as ligands for prey recognition by the mycoparasite and induce the production and mass release of cell wall degrading enzymes and other associated cellular processes (Brunner et al., 2003; Benítez et al., 2004; Viterbo et al., 2007; Druzhinina et al., 2011). This cascade of events is considered as the starting point for the activation of a mycoparasitic attack program in various *Trichoderma* species (Zeilinger et al., 1999; Viterbo et al., 2002; Brunner et al., 2003). However, little is known about the prey-derived molecules that trigger the directed growth of mycoparasitic hyphae toward those of the prey fungus (Harman et al., 2004; Vinale et al., 2008). Chemotropic events, that either lead to positive chemotropism guided by compounds that elicit attraction (Lichius and Lord, 2014), or to negative chemotropism, when a compound has a repellent or toxic activity (Miyoshi, 1894), results in hyphal tropism. Most of these observations have been made by evaluating the growth redirectioning of conidial germlings from fungi like *Penicillium glaucum*, *B. cinerea, Trichoderma harzianum* or *F. oxysporum*, toward gradients of self-generated or exogenously derived chemical substances to test their chemotropic activity (Miyoshi, 1894; Massee, 1905; Turrà et al., 2015; Vitale et al., 2017, 2019; Lombardi et al., 2018; Partida-Hanon et al., 2020). Chemotropism in hyphae has been investigated under different conditions, including the presence of potentially harmful products (negative chemotropism), or autotropism, comprising chemical attraction between genetically identical cells by means of vegetative hyphal fusion (Hickey et al., 2002; Roca et al., 2005) or self- avoidance mediated by low oxygen content areas (Brand and Gow, 2009). Sexual attraction is an essential non-self signaling process on the basis of secreted mating type-specific peptide pheromones (Gooday and Adams, 1993; Turrà and Di Pietro, 2015), and represents the so far best understood chemotropic mechanism.

How the perception of diffusible compounds released by plant hosts triggers morphological responses in fungal plant pathogens that ultimately lead to parasitism are crucial insights required to broaden our understanding of the physiological and molecular factors involved in this process (Brand and Gow, 2009; Turrà et al., 2015, 2016; Nordzieke et al., 2019). Proof of these chemotropic events, however, often remains elusive due to the lack of proper evaluation schemes.

In this study, we have developed three different chemotropic assays to evaluate the chemical sensing ability of the mycoparasite *T. atroviride* toward selected compounds known or suspected to play essential roles in the mycoparasite/plant host/prey fungus interaction covering the complete spectrum of the fungal developmental stages from conidial germling to a mature colony. This comparison revealed that during all developmental stages association with the plant host and self-signaling for colony network establishment are prioritized, while fungal prey recognition becomes increasingly important as the colony matures. These findings suggest that germling assays are highly useful for rapid screening of chemotropic compounds, while the analysis of fully differentiated colonies is essential for the identification of chemotropic compounds that are specific for a given process such as plant symbiosis or mycoparasitism.

## Materials and Methods

### Strains and Culture Conditions

*Trichoderma atroviride* strain P1 (ATCC 74058) was used as mycoparasite; *B. cinerea*, *R. solani, Sclerotinia sclerotiorum, F. oxysporum* f. sp. *lycopersici*, and *F. graminearum* PH-1, were used as prey fungi. *B. cinerea* is a plant leaf pathogen, whereas *R. solani* and the two *Fusarium* species attack plant roots and spikes, respectively. Pre-cultures of all strains were grown on potato dextrose agar (PDA) (BD Difco, Franklin Lakes, NJ, United States), and incubated for 7 days at 22°C for *B. cinerea* and at 25°C for all other fungal strains, under a 12/12 h light/dark regime.

Liquid cultures for supernatant production were set up using M9 minimal medium (M9, per l: 64 g Na_2_HPO_4_-7H_2_O, 15 g KH_2_PO_4_, 2.5 g NaCl, 5 g NH_4_Cl, 2 ml 1M MgSO_4_, 100 μl 1M CaCl_2_, 15 g agar for solid medium, pH 5.5) with 2% w/v glucose, and inoculated with 1 ml of 10^6^ conidia/ml of either *T. atroviride, B. cinerea* or *F. oxysporum*, and in the case of *R. solani* with small agar plugs (5 mm^3^) carrying mycelium. Liquid cultures of a total volume of 75 ml were incubated for 72 h at 25°C using shaking at 200 rpm. Liquid cultures for fungal cell wall extraction were set up accordingly but using malt extract medium (MEX) (VWR Chemicals BDH, Radnor, PA, United States) instead of M9.

### Antagonistic Activity of *Trichoderma* (Mycoparasitism)

Dual-confrontation assays between *T. atroviride* and *B. cinerea*, *R. solani*, *F. oxysporum*, and *F. graminearum*, respectively, were performed by placing small agar blocks carrying mycelium of the mycoparasite and the prey fungus 6 cm apart from each other on PDA plates, and incubating the co-culture for 7 days at 22°C for *B. cinerea* and 25°C for all other fungal strains, under a 12/12 h light/dark regime. All assays were performed in triplicates and repeated at least one time.

### Fungal Biochoice Assay (Multiple Confrontation Assay)

**FIGURE 1:**
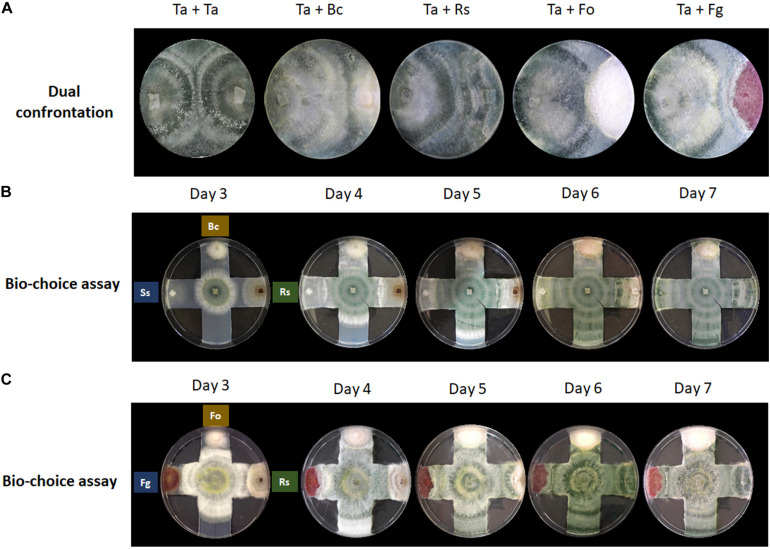
Confrontation assays provide no indication for prey preferences of *T. atroviride.* **(A)** Dual confrontation and **(B,C)** biochoice assays between *T. atroviride* (Ta), *B. cinerea* (Bc), *R. solani* (Rs), *S. sclerotiorum* (Ss), *F. oxysporum* (Fo), and *F. graminearum* (Fg).

Biochoice assays were performed as previously described (Gerardo et al., 2006) with slight modifications. Large Petri dishes (14 cm diameter) filled with 50 ml PDA were cut out in crux form to create four 4-cm-wide tracks (Figure 1). *T. atroviride* was inoculated in the center, and each track was assigned to one condition: control (empty track), track 1, track 2, and track 3. The five prey fungi were divided into two sets in order to consistently maintain the same potential inter-species effects in all confrontations against *T. atroviride*. Set one comprised *S. sclerotiorum, B. cinerea*, and *R. solani*; set two comprised *F. graminearum*, *F. oxysporum*, and *R. solani.* Small (5 mm^3^) agar blocks carrying mycelium of either the mycoparasite or the prey fungi were used for inoculation. While *B. cinerea*, *F. graminearum*, and *F. oxysporum* were inoculated 1 day in advance to *T. atroviride* to take different growth rates into account, *S. sclerotiorum* and *R. solani* were inoculated simultaneously with *T. atroviride*. The cultures were incubated at 25°C under a 12/12 h light/dark regime and colony development was documented photographically from day 3 to 10. All assays were performed in triplicates and repeated at least one time.

### Filtration and Molecular Size Fractionation of Liquid Culture Supernatants

Supernatants of liquid cultures were obtained by filtration through a double layer of Miracloth (Merck Millipore, Burlington, MA, United States). Fractionation of supernatants was performed using Amicon Ultra 15 ml centrifugal filters (Merck Millipore, Burlington, MA, United States) with molecular weight cut-offs of 3, 10, 30, and 100 kDa, respectively, according to manufacturer’s recommendations.

### Cell Wall Extraction

Total cell wall extract (CWE) from *T. atroviride, B. cinerea, F. oxysporum*, and *R. solani* were prepared from liquid cultures. Fungal biomass was separated from MEX medium by filtration through a double layer of Miracloth (Merck Millipore, Burlington, MA, United States). The biomass was washed twice with distilled water, dried at 50°C overnight, and weighed. Two grams of dried mycelium were added to 100 ml of 96% ethanol and boiled for 30 min to extract low molecular weight solutes. The remaining insoluble material was separated using filter paper (grade 3 m/N, 65 g/m^2^) (Ahlstrom Germany GmbH, Art. No. 2.305.580580, Bärenstein, Germany) and washed with 200 ml of chloroform:methanol (1:1, v/v) and 200 ml of acetone to yield the crude cell wall preparation (alcohol insoluble solids, AIS). Total cell wall was dried overnight at 37°C, and then stored at room temperature until further use.

### Chemotropism Assay With Conidial Germlings

The previously described chemotropism assay (Turrà et al., 2015) was adapted for *T. atroviride*. The selected medium was a modified M9 (MM9) minimal medium with reduced nitrogen (26.745 mg/l NH_4_Cl) and carbon (18.016 mg/l glucose) source content. Microconidia were harvested from 6-day old *T. atroviride* PDA cultures and diluted to a final concentration of 2.5 × 10^6^ cells per ml. 100 μl of the adjusted conidial suspension were spread on MM9 in standard-size Petri dishes using glass beads. A central scoring line was drawn on the bottom of the plate, and two circular filter papers of 1.2 cm diameter previously inoculated with 25 μl of the test compound solution or solvent control were placed with 5 mm distance on either side of the scoring line. In gradient competition experiments, control solution and test compound were applied to either filter paper.

Tested compounds and their standard concentrations were: glucose (Gluc), copper sulfate pentahydrate (CuSO_4_^∗^5H_2_O), manganese chloride (MnCl_2_), and zinc chloride (ZnCl_2_), all at 10 mM; sodium glutamate (Glu), glutamine (Gln), methionine (Met), all at 295 mM; ammonium nitrate (NH_4_NO_3_), galactose (Gal), and N-acetyl-D-glucosamine (NAG), all at 50 mM; cellulose (Cel) at 1% (w/v); 6-amyl-α-pyrone (also known as 6-pentyl-2-pyrone, 6-PP) (Sigma-Aldrich, CAS number 27593-23-3, Buchs, Switzerland) at 10 μM or 10 mM, and 10E, 12Z-9S-9-Hydroperoxyoctadeca-10, 12-dienoic acid (9(s)-HpODE), 9Z,11E,13S-13-hydroperoxyoctadeca-9,11-dienoic acid (13(s)-HpODE), 9-hydroxy-10E, 12Z-octadecadienoic acid (9(s)-HODE), and 13S-hydroxy-9Z,11E-octadecadienoic acid (13(s)-HODE) (Cayman Chemical, CAS numbers: 29774-12-7, 33964-75-9, 73513-67-6, 29623-28-7, respectively, Michigan, United States), all at 10 mM; α-mating factor from *Saccharomyces cerevisiae* (Sigma-Aldrich, CAS number T6901. Buchs, Switzerland) at 378 μM; root exudate from *Solanum lycopersicum* [prepared as described in Turrà et al. (2015)], liquid culture supernatants of *T. atroviride* (SnTa), *B. cinerea* (SnBc), *F. oxysporum* (SnFo), and *R. solani* (SnRs), and variants thereof supplemented with CWE from *T. atroviride, B. cinerea*, and *R. solani*, respectively.

Sterile water or ethanol were used as solvent controls. Conidial germ tube development was evaluated after 14 h of incubation at 25°C. The chemotropic index was calculated as (CI = (H_test_ – H_solv_)/H_total_ × 100) (Turrà et al., 2015), where H_test_ is the number of germ tubes growing toward the test compound, H_solv_ is the number of germ tubes growing toward the solvent control, and H_total_ is the total number of counted germlings. For each test compound a total of 100 germ tubes were scored. All assays were performed in triplicates and repeated at least one time. Statistical analysis was conducted using *t*-test.

### Chemotropism Assay With Microcolonies

Four inoculation points, each separated by 1.2 cm, were defined along an outer perimeter of a MM9 agar plate. One microliter conidial suspension with either 10 or 100 cells per μl was placed on each inoculation point. Circular filter paper of 1.2 cm diameter soaked with 25 μl of the test compound solution or solvent control was placed at the center of the plate right after inoculation. Cultures were incubated at 25°C and a 12/12 h light/dark regime and colony development was photographically and microscopically evaluated after 24 and 36 h. All assays were performed in triplicates and repeated at least one time.

### Chemotropism Assay in Hyphal Growth Format

Solid MM9 in standard Petri dishes was cut in “T” form to create a 1 cm wide and 3.5 cm long agar track that limits lateral colony expansion and forces colony development toward the test area of the culture plate. A small agar plug (5 mm^3^) carrying non-sporulating mycelium of *T. atroviride* was placed in the middle of the track and incubated at 25°C for 36 h or until the colony reached the scoring line. Two 1.2 cm diameter filter paper discs soaked with 25 μl of test compound solution or solvent control were placed on either side of the scoring line at a 5 mm distance. The culture was incubated for an additional 14 h before preferential growth direction and covered area of colony expansion beyond the scoring line were evaluated, and photographically and microscopically documented. All assays were performed in triplicates and repeated at least one time.

### Statistical Analysis

Data were subject to one-way analysis of variance (ANOVA). By using least significance difference (LSD) at *p* = 0.05, treatment means were separated (Saville, 1990). *Post hoc* contrast (Scheffé) analysis was used to explore differences between group means by using least significance difference at *p* = 0.05 (Scheffé, 1959). All analyses were performed using the package IBM SPSS Statistics 24.

## Results

### Response of Trichoderma atroviride to Fungal Prey Stimuli and Mycoparasitic Behavior

Confrontation assays with *T. atroviride* are most frequently performed in a one-to-one format to evaluate the mycoparasitic behavior in response to a single prey fungus (Figure 1A). This classic assay format is highly valuable to gain new insights at the macroscopic level into general attack, defense and counter-attack responses displayed by mycoparasite and prey. Certain plant pathogens, such as *B. cinerea* and *R. solani*, are easily mycoparasitised by *T. atroviride*, i.e., after 7 days under standard confrontation conditions as previously described (Zeilinger et al., 2005). By contrast, other plant pathogens such as *F. oxysporum* or *F. graminearum* are very difficult to almost impossible to be mycoparasitised by *T. atroviride* under standard assay conditions, since the mycoparasite fails to overgrow the prey mycelium or does so only to a small extent (Speckbacher et al., 2020b).

The hypothesis that each prey fungus displays different defense mechanisms against the mycoparasitic attack by *T. atroviride* prompted us to investigate if *T. atroviride* has a differential chemotropic behavior toward its preys. To this aim, multiple confrontation assays were conducted to evaluate changes in growth direction, growth speed, and mycoparasitic efficiency of *T. atroviride* in response to different prey fungi (Figure 1). We noted that *T. atroviride* did not show significant changes in speed or direction of hyphal growth, nor did we observe specific mycoparasitism-associated morphological changes related to individual prey fungi, except in the interaction with the two *Fusarium* species which it was unable to overgrow. This suggests that neither the classic confrontation assay nor the biochoice assay is suitable for revealing particular attack preferences or subtle changes in colony morphology in response to different prey species. Hence, the screening of potential chemotropic compounds relevant to mycoparasitism needs to be performed with a different assay format. Furthermore, the classic fungus-fungus confrontation assay completely excludes the plant host, which is an essential symbiotic partner in the natural context of the rhizosphere. Therefore, we decided to examine the response of *T. atroviride* germlings to different chemotropic compounds relevant to plant symbiosis and mycoparasitism.

### Minimum Carbon and Nitrogen Quantities, pH Stability and Increased Oxygen Availability Are Essential for Stress-Free Chemotropism Assays With *Trichoderma atroviride*

**FIGURE 2:**
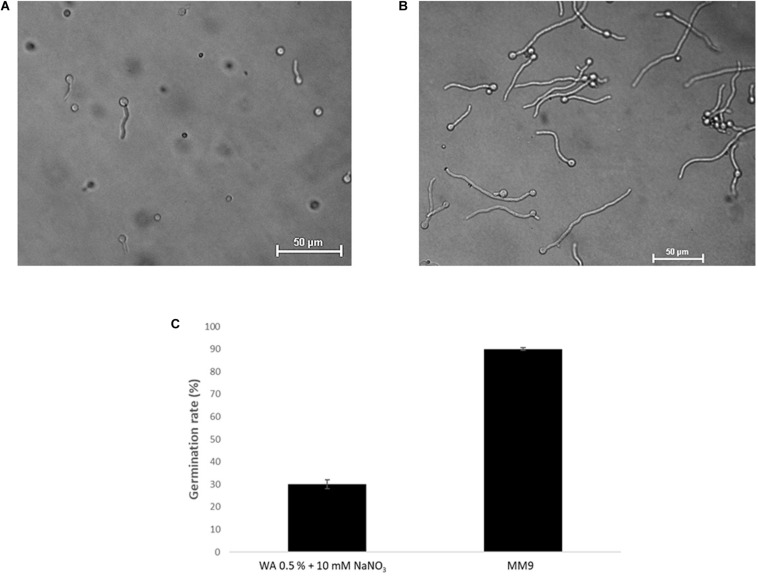
Optimization of chemotropism assays for *T. atroviride*. **(A)** Germlings of *T. atroviride* displaying stress morphology after 14 h incubation in water agar (WA). **(B)** Germlings of *T. atroviride* displaying healthy morphology after 14 h incubation on MM9 agar. **(C)** Supplementation of WA with 10 mM NaNO_3_ rescued normal germling morphology, but restricted the total germination rate to no more than 30%, whereas MM9 with 0.1 mM glucose and 0.5 mM NH_4_Cl maximized the germination rate of healthy germlings to up to 90%.

**FIGURE 3:**
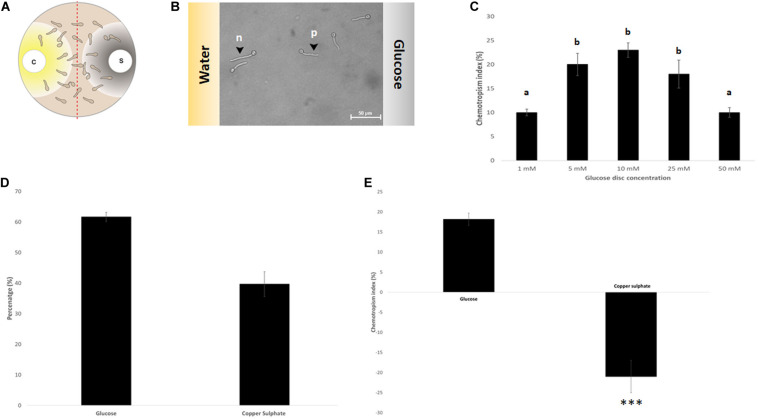
Assay verification by evaluating the chemotropic index of controls. **(A)** Schematic representation of the double diffusion solvent (S)-compound (C) assay on MM9 plates. **(B)** Germlings of *T. atroviride* after 14 h incubation directing their growth toward glucose (p = positive chemotropic response) and toward water (n = negative chemotropic response). **(C)** Titration of the glucose control from 1 to 50 mM identified 10 mM as the optimum for chemoattraction. **(D,E)**. Evaluation of chemoattraction and chemorepulsion as percentage of the total cell population **(D)**, or as chemotropic index (CI = (H_test_ − H_solv_)/H_total_ × 100) **(E)**. Different letters indicate significant differences between the different glucose concentrations at *p* < 0.05 using Scheffé test (^∗∗∗^*p* ≤ 0.001).

Based on an assay design previously used to quantify the chemotropic response in *F. oxysporum* germlings (Turrà et al., 2015), the experimental conditions were optimized for *T. atroviride* to enable detection of chemotropic responses in a stress-free environment. The use of embedded growth in water agar – as used by Turrà and co-workers for *F. oxysporum* – produced abnormally shaped germlings and reduced the germination rate of *T. atroviride* to only 20–30%, two features indicating significant cell stress (Figure 2 and Supplementary Figure S1). Addition of at least 27 mM glucose and 10 mM NaNO_3_ complemented germ tube morphology but failed to increase germination rate (Figure 2). This suggests that starvation stress was alleviated but that other abiotic stresses remained present. Therefore, water agar was replaced with a pH-buffered MM9. After titrating increasing molarities of glucose and ammonium chloride as the sole carbon (C-) and nitrogen (N-) sources, respectively, 0.1 mM glucose and 0.5 mM NH_4_Cl were found to optimize the morphological appearance of germlings and to improve overall chemotropic behavior (Supplementary Figure S2). The intention was to supply as much C- and N-sources as necessary for healthy germling development, but as little as possible to avoid nutrient sensing interfering with chemotropic processes related to the perception of chemotropic cues derived from plant host or prey fungus. In addition, conidia were not embedded into the agar but instead homogeneously spread on top using glass beads, in order to ensure maximum oxygen availability and to facilitate microscopic observation. Taken together these measures improved the germination rate of healthy growing germlings to the experimentally achievable maximum of 80–90%. Only germlings that developed germ tubes of at least 50 μm length under these conditions were considered for quantification of chemotropism. Before adding test chemoattractant compounds, initial verification of the method was performed by double diffusion assays with glucose as positive and copper sulfate as negative chemotropic controls. From a tested range of 1–50 mM glucose as starting concentration on the filter disc, 10 mM provided the strongest chemoattractive stimulus, whereas the initial application of 10 mM CuSO_4_ was consistently chemorepellent (Figure 3). Chemotropic behavior can be quantitatively expressed as simple percentage of the total number of evaluated germlings or as chemotropic index (CI) (Turrà et al., 2015). The comparison of both scoring methods showed that the percentage expression does not clearly illustrate the difference between chemoattraction and chemorepulsion (Figure 3). Hence, CI calculation was used as the scoring method of choice in this study.

Initial tests confirmed that positive chemotropism/chemoattraction could be quantitatively evaluated in a concentration-dependent manner and it could be clearly differentiated from negative chemotropism/chemorepulsion. Therefore, the basic assay design was deemed ready for screening additional test compounds.

### *Trichoderma atroviride* Germlings Show Differential Chemotropic Responses to a Variety of Chemical Compounds

Test compounds were divided into three groups representing: (1) elementary nutrients and toxins, (2) elementary self and non-self signaling compounds, and (3) complex self and non-self signaling compounds. The major aims of this set of experiments were to consolidate positive and negative controls, identify the most active non-nutrient chemoattractants for *T. atroviride* relevant in the context of plant symbiosis and mycoparasitism, and to collect the initial biochemical evidence to identify plant hosts or fungal prey species that secrete chemoattractants for *T. atroviride*.

**FIGURE 4:**
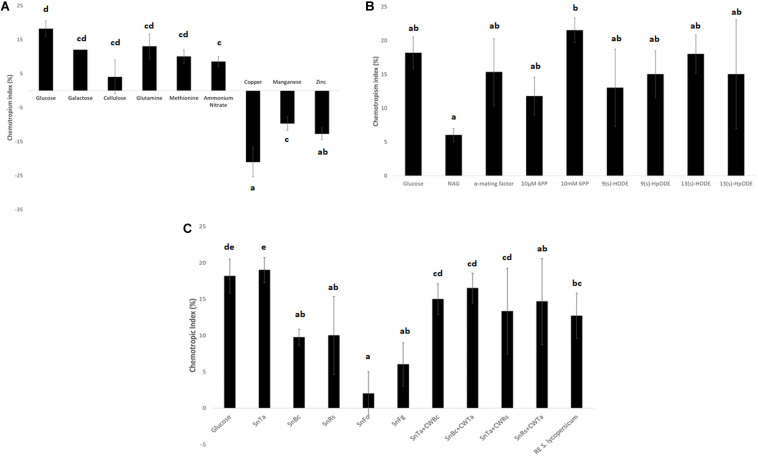
Chemotropic responses to different classes of test compounds. **(A)** Elementary nutrients and toxins: glucose and copper were confirmed as highly chemoattractive and chemorepellent control compounds for *T. atroviride*. Furthermore, the assay reflects the declining cellular preference for the compared C- and N-sources with decreasing CI values (see text for details). **(B)** Elementary self and non-self signaling compounds: 10 mM 6PP of *T. atroviride* and the plant oxylipin 13(s)-HODE stand out as the most potent chemoattractants for *T. atroviride*, with the highest, yet concentration dependent, positive response to 6PP. **(C)** Complex self and non-self signaling compounds: in analogy to 6PP, SnTa provides the strongest chemoattractive cue to conidial germlings of *T. atroviride*. This is followed by root exudate (RE) of *Solanum lycopersicum*. Furthermore, does the assay reflect the distinction between prey fungi which can easily or hardly be overgrown by *T. atroviride*, with SnBc and SnRs resulting in much higher CI values than SnFo and SnFg. Liquid culture supernatants generated in the presence of total cell walls extracts (CWE) of another fungal species shifted the CI values in different directions in comparison to Sn alone (see text for details). Different letters indicate significant differences between the different compounds at *p* ≤ 0.05 using Scheffé test.

The comparison of three different carbon sources confirmed that glucose was the strongest chemoattractant (Figure 4A). This result is not surprising, as it is the easiest to be metabolized and thus the preferred C-source. Likewise, the energetically preferred N-source glutamine produced the second highest positive CI value after glucose. In contrast, copper produced the highest negative CI value compared to zinc and manganese, confirming its status as a potent chemorepellent (Figure 4A). Due to the main interest in chemoattraction and the lack of a non-toxic, chemorepellent control for *T. atroviride*, we exclusively used glucose as positive control in all subsequent assays.

Elementary self-signaling compounds tested comprised N-acetyl-glucosamine (NAG), an important inducer of chitinase production in *T. atroviride* (Brunner et al., 2003), and 6-PP, an antifungal low molecular weight compound produced by *T. atroviride*. Tested non-self signaling compounds included four different plant oxylipin variants. 10 mM 6-PP, *S. cerevisiae* α-mating factor, and the plant oxylipin 13(s)-HODE were identified as the most potent chemoattractants for *T. atroviride* germlings (Figure 4B). The attraction to plant stress signals makes sense in terms of establishing a symbiosis and as chemical cues toward the location of a potential prey fungus in the rhizosphere.

Complex self and non-self signaling compounds tested comprised liquid culture supernatants of *T. atroviride* (SnTa), of the two easily overgrown prey species *B. cinerea* (SnBc) and *R. solani* (SnRs), and of the two less parasitized species *F. oxysporum* (SnFo) and *F. graminearum* (SnFg). Furthermore, supernatants of *T. atroviride* cultures supplemented with CWEs of other fungal species were included as well as tomato root exudate. In analogy to 6-PP, SnTa elicited the strongest chemotropic response in conidial germlings of *T. atroviride*, followed by tomato root exudate (Figure 4C). Furthermore, the assay reflected the distinction between prey fungi that could be overgrown easily or with difficulty by *T. atroviride* in the confrontation assay (see Figure 1), with the supernatants of *B. cinerea* and *R. solani* resulting in much higher CI values than those of the two *Fusarium* species (Figure 4C). Supernatants of cultures containing CWEs from another fungal species had differential effects. The general trend was that addition of CWEs from prey species decreased the CI of SnTa, whereas the addition of CWTa increased the CI of prey species Sns. This indicates that cell wall components of *T. atroviride* present higher self-chemoattractive cues than cell wall components from prey fungi in terms of non-self chemoattraction of *T. atroviride* germlings.

Taken together these data suggest that conidial germlings of *T. atroviride* are most perceptive toward self-signaling compounds released by other germlings and hyphae of *T. atroviride*, as well as to non-self signaling compounds released by plant roots. The underlying biological sense is most likely linked to the need for rapid colony establishment and symbiotic association with a plant partner. The relevance of non-self chemoattraction to other fungi might become more important at later developmental stages, when a proper colony network of mature hyphae has been established, that are capable of mass secretion of cell wall degrading enzymes. To test this hypothesis, we next used microcolonies of *T. atroviride* as chemoresponse screening targets.

### Microcolonies of *Trichoderma atroviride* Locally Increase Hyphal Density in Response to Chemotropic Cues

So far there is no evidence for mycoparasitic activity in conidial germlings of *T. atroviride* or other species. Based on the definition that the appearance of a Spitzenkörper marks the transition from germ tube to hypha (Araujo-Palomares et al., 2007), mycoparasitism is likely to be an exclusive function of the latter. The next logical step, therefore, was to evaluate morphological responses of *T. atroviride* hyphae in chemotropism assays using microcolonies. Microcolonies represent a transitional stage between the conidial germling network and the fully differentiated mature fungal colony. Microcolonies of *T. atroviride* developed up to an average diameter of about 1–2 cm, before the differentiation of leading hyphae, primary and secondary branches began and functional separation between sub-periphery and periphery commenced. Until that transition, all hyphae appeared similar with respect to average diameter, branching and hyphal fusion patterns (data not shown).

**FIGURE 5:**
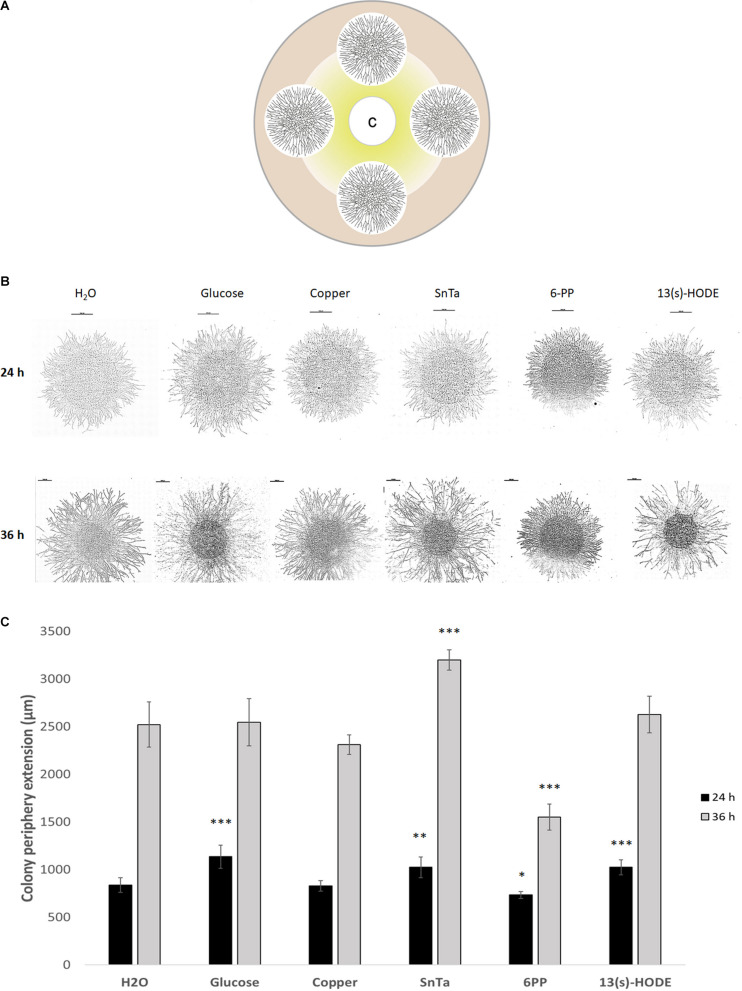
Microcolonies of *T. atroviride* respond differentially to chemoattractive and chemorepellent compounds by altering colony extension rate and hyphal density. **(A)** Schematic representation of the assay design, showing four fungal microcolonies of about 1.2 cm diameter arranged equidistant to each other and the central filter disc containing the test compound C. From the filter disc, radial diffusion creates a compound gradient indicated in yellow. **(B)** Tiled composite microscopic images of microcolonies that developed distinct morphologies with respect to colony extension rate and hyphal density after 24 h and 36 h exposure to the indicated compounds. Scale bars, 1 mm. **(C)** Quantification of average colony extension rates of microcolonies exemplified in panel **(B)**. Left bars after 24 h compound exposure, right bars after 36 h. SnTa stands out as the most effective modifying compound, however, 6PP and 13(s)-HODE also show significant effects; ^∗^*p* ≤ 0.05, ^∗∗^*p* ≤ 0.01, ^∗∗∗^*p* ≤ 0.001.

Still, distinct morphological responses to positive or negative chemotropic compounds were detected at this stage. Glucose, for instance, increased colony extension and hyphal density, whereas copper restricted colony extension and hyphal density (Figure 5). SnTa and 13(s)-HODE most notably promoted colony extension, while 6-PP most notably led to an increase in hyphal density combined with a reduction of the colony extension rate. The longer the exposure time, the more pronounced these features developed. Together, these results confirm that chemoattractants are strongly growth stimulating. The comparison furthermore shows that – at least at this early stage of colony development – sensing and signaling in *T. atroviride* is more sensitive toward self and plant cues than to non-self cues.

### *Trichoderma atroviride* Redirects Colony Extension Locally Toward Positive Chemotropic Compounds

**FIGURE 6:**
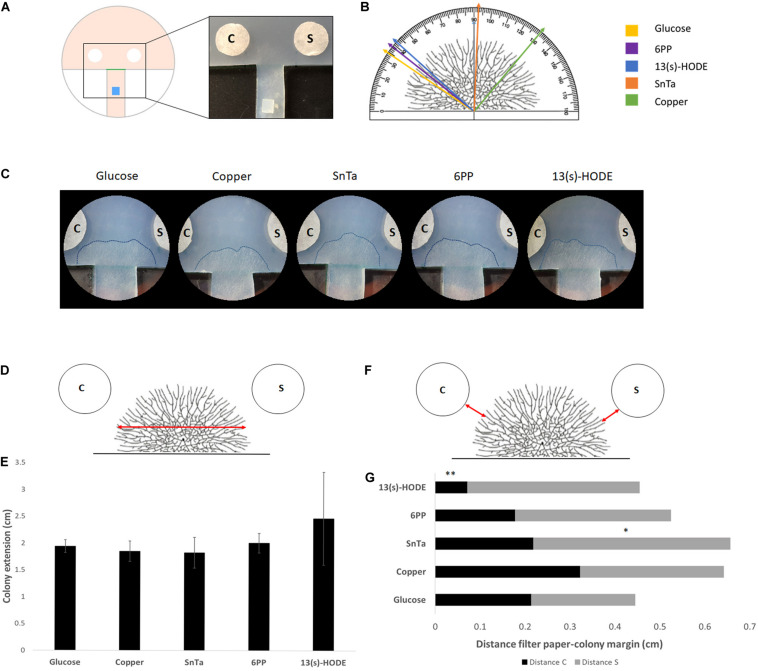
Expansion-limited chemotropism assay with area-restricted colonies of *T. atroviride* reveal localized morphological responses of the colony periphery to distinct chemotropic cues. **(A)** Schematic representation of the assay design. The inoculum (blue) is placed on an agar track that forces colony development toward the test area between the test compound and solvent control filter discs. The timing, preferential growth direction and covered area of colony expansion are determined from the score line (green). The enlarged image shows the corresponding region of an actual culture plate. **(B)** Representation of a *T. atroviride* colony and different approach angles (colored arrows) that indicate how colony edge extension shifts after 14 h exposure to the different compounds. **(C)** Representative examples of expansion-limited double diffusion solvent control (S)/test compound (C) assays on MM9. The outer margins of developed *T. atroviride* colonies after 14 h incubation in the presence of the compound gradient are outlined (dashed lines). Localized colony chemoattraction is considered when hyphal density and colony edge extension increase and shift toward the compound. **(D,E)** Representation of *T. atroviride* colony and transversal red arrow indicating measurement of colony expansion **(D)** and colony extension **(E)** after 14 h exposure to different compounds. **(F,G)** Representation of *T. atroviride* colony and red arrows indicating distance between colony margin and inoculation point **(F)** and distance measurements from compound (C) and solvent (S) **(G)** after 14 h exposure to different compounds. ^∗^*p* ≤ 0,05, ^∗∗^*p* ≤ 0,01.

The discernible morphological adaptations in response to chemotropic compounds were quickly lost as the microcolony expanded. Hence, in order to increase the spatio-temporal resolution of the chemotropic growth behavior of fully differentiated colonies, the available substrate area was artificially limited by preparing a defined growth track that restricts lateral colony expansion and directs a small sector of the colony periphery to a non-restricted area between the test compound and the solvent control (Figure 6A). This allowed the differentiation of further advanced developmental colony stages while preventing hyphal congestion, which would otherwise obstruct the resolution of subtle compound-induced morphological changes close to the sources.

With this set up, we confirmed that defined regions of the colony margin focused growth toward chemoattractants and away from chemorepellents (Figures 6B,C). The most effective chemoattractants in this assay format were 6-PP and 13(S)-HODE, indicating that fully differentiated, mature colonies of *T. atroviride* are still sensitive to self-signaling while becoming more sensitive to non-self signaling, represented by a known plant stress compound (Figures 6D–G).

Sensing of 13(s)-HODE seemed to increase growth speed, as indicated by the significantly reduced distance between colony margin and inoculation point in comparison to the glucose control. This result further supports the notion that *T. atroviride* is most affected by plant stress signals during later developmental stages.

### Fractionation of Culture Supernatants Results in Up to Four-Times Increased CIs

**FIGURE 7:**
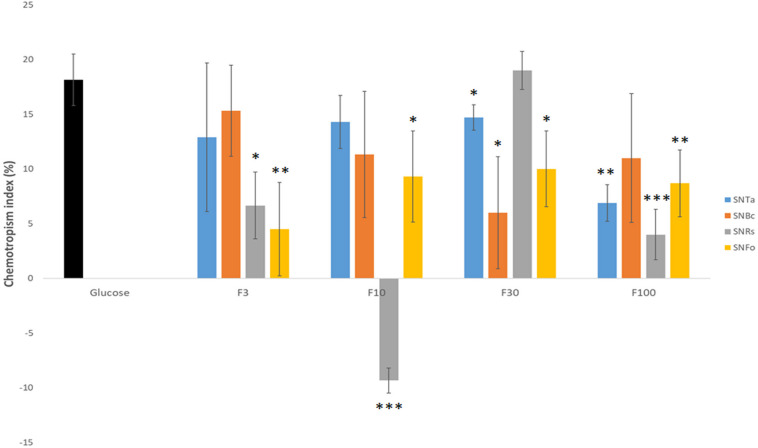
Molecular weight-fractionation of culture supernatants improves CI resolution. Chemotropic responses to culture supernatants of *T. atroviride* (SnTa), *B. cinerea* (SnBc), *R. solani* (SnRs), and *F. oxysporum* (SnFo). Outstanding are the increased CI-values for F3 of SnBc, F30 of SnRs, and F10 and F30 of SnFo, which are two- to four-times higher than the CIs of the corresponding non-fractionated Sns. This shows that molecular-size fractionation removes equalizing effects between chemoattractants and chemorepellents combined in the crude mixtures, and, furthermore, suggests that the chemoattractant molecules responsible for prey-specific responses of *T. atroviride* are enriched in specific fractions. ^∗^*p* ≤ 0.05, ^∗∗^*p* ≤ 0.01, ^∗∗∗^*p* ≤ 0.001.

The concentration of fungal prey-derived chemoattractants in the complex SnBc, SnRs and SnFo mixtures was apparently too low to induce morphological changes visible at the macroscopic and low-magnification microscopic levels. Therefore, molecular size-separated fractions of culture supernatants were prepared and responses in germling, microcolony and expansion-limited chemotropism assays evaluated. The > 3, > 10, > 30 and > 100 kDa fractions (F3 – F100) from liquid culture supernatants were used as test compounds in double-diffusion germling assays. F10 and F30 of the *R. solani* supernatant (SnRs) induced the highest negative and positive chemotropic responses in *T. atroviride* germlings, reaching CIs of −10% and + 20%, respectively (Figure 7). Notably, the CI of SnRs F30 had a two-fold higher chemoattractant effect than the non-fractionated SnRs (see Figure 4), suggesting that in the crude mixture chemoattractants and chemorepellents equilibrate each other. Furthermore, this experiment indicates that the molecule(s) attracting *T. atroviride* to its prey *R. solani* are in the molecular weight range between 30 and 100 kDa. The results with SnBc F3 suggested a similar role for molecules < 3 kDa from *B. cinerea*. Fractionation of SnFo resulted in an up to four-times higher positive CI compared to the non-fractionated mixture, which was almost evenly distributed across F3 – F100, with no particular fraction standing out. Interestingly, no strongly chemorepellent fraction emerged from SnFo, indicating that the difficulty of *T. atroviride* to parasitize *F. oxysporum* is not due to chemorepulsion, but rather to a highly effective defense response of *F. oxysporum*. In contrast to the fractionated prey supernatants, the CI values of SnTa F3-F100 were at least 5% lower compared to the non-fractionated SnTa (Figure 7), indicating that auto-attraction may require a mixture of molecules from different size ranges. Nevertheless, fractions triggering higher CI values than the glucose control (SnRs F30) or strongly negative CIs (SnRs F10) were considered top candidates for further evaluation.

### Sn Fractions Trigger Enhanced Chemotropic Responses in Microcolonies

**FIGURE 8:**
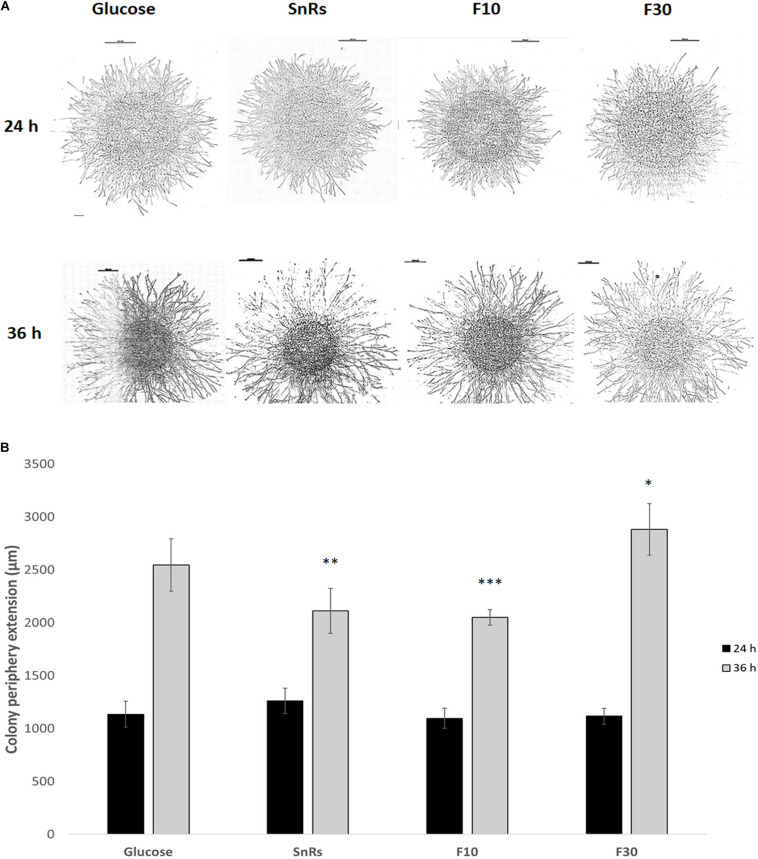
Chemotropism assay with microcolonies of *T. atroviride*. **(A)** Microscopic images of microcolonies that developed different colony morphologies including hyphal density in response to 24 h (top row) and 36 h (bottom row) compound exposure, respectively. Scale bars, 1 mm. **(B)** Quantification of the average colony periphery extension showing that it is more affected after 36 h exposure to the selected compounds, especially in the presence of the SnRs F10 fraction. Hence, the significant chemotropic influence SnRs F10 and F30 have on conidial germlings also shows in the altered morphological development of microcolonies. ^∗^*p* ≤ 0.05, ^∗∗^*p* ≤ 0.01, ^∗∗∗^*p* ≤ 0.001.

Microcolony chemotropism assays (see Figure 5A) were conducted to test whether Sn fractionation has similar effects on microcolony development as on germling chemotropism. After 24 h exposure, SnRs F10 restricted colony extension in comparison to the glucose control, the crude SnRs and SnRs F30 (Figure 8). Six hours later, hyphal density in the sample decreased, whereas it significantly increased in the presence of SnRs F30. This antagonistic effect of SnRs F10 and F30 fully reflects the results obtained with conidial germlings, although morphologically the effects in the microcolony were visible as an alteration of colony extension rate and hyphal density.

### Directional Colony Expansion Is the Only Discernible Feature Affected by Sn Fractionation in Mature Colonies

**FIGURE 9:**
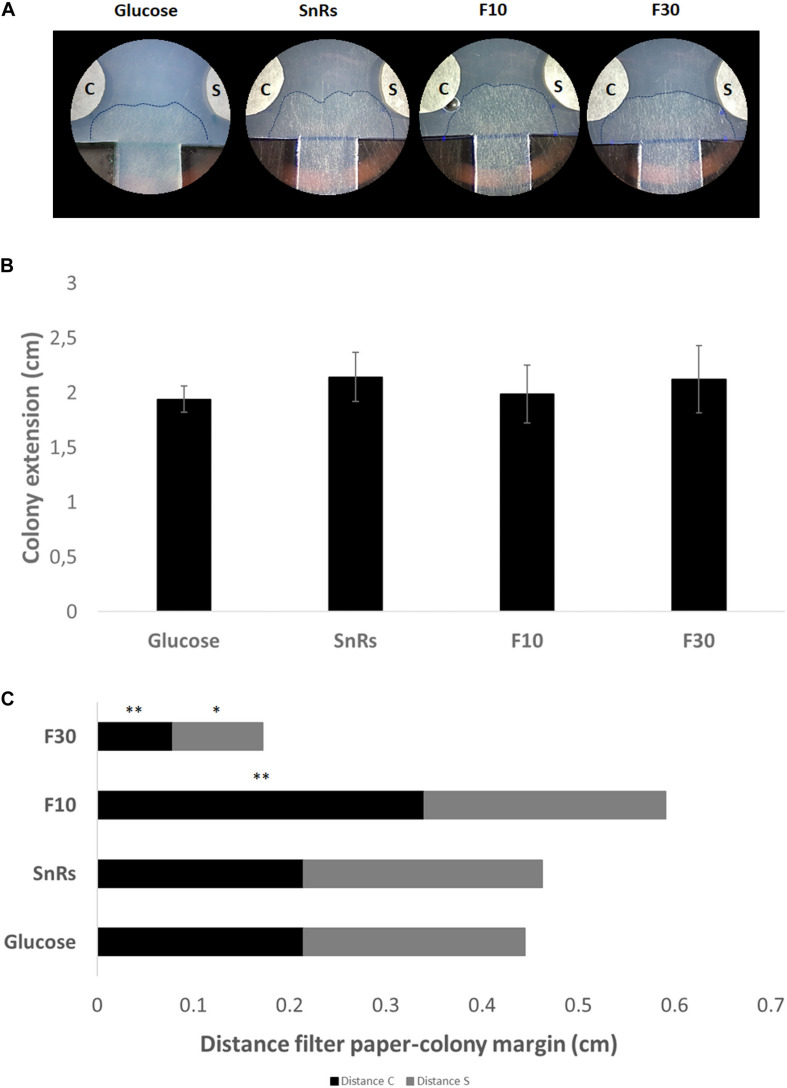
Expansion-limited chemotropism assay with area-restricted colonies of *T. atroviride*. **(A)** Representative examples of double diffusion solvent (S)/test compound (C) assays on MM9. The outer margins of developed *T. atroviride* colonies after 14 h incubation in the presence of the compound gradient are outlined (dashed lines). Colony chemoattraction is observed as colony edge extension increase when fractions are tested. **(B)** Colony extension measurements after 14 h exposure to SnRs, its F10 and F30 fractions, and the glucose control, respectively. **(C)** Distance measurements from compound (C) and solvent (S) after 14 h exposure to fraction 10 (F10) and 30 (F30) of SnRs. Both fractions show opposite effects: F30 colony margins increase toward the compound while F10 limits the colony margin growth that correlates with CI obtained in the chemotropism assay with germlings. ^∗^*p* ≤ 0.05, ^∗∗^*p* ≤ 0.01.

Expansion-limited chemotropism assays with the SnRs fraction revealed that the alterations of overall colony expansion rate or hyphal density seen in microcolonies were no longer visible in the mature colony (Figure 9). Directional expansion and localized growth inhibition, nevertheless, indicated that the colony periphery is still susceptible to chemotropic cues (Figure 9C). SnRs F30, for instance, increased expansion of the colony periphery toward the compound source within less than 14 h of exposure, whereas the chemorepellent SnRs F10 prevented the colony periphery from reaching the compound source in the same time period. The crude SnRs mixture gave intermediate results, while the glucose control confirmed the positive effect of the F30 fraction. These results support the idea that mature hyphae of the fully differentiated colony periphery are the primary sensors for and responders to external chemotropic cues, while hyphae constituting the sub-periphery fulfill other tasks.

## Discussion

The results of our study indicate that chemotropic compounds can elicit distinct morphological responses in *T. atroviride*, whose phenotype changes as the fungus develops from germling to mature colony. Conidial germlings respond by either directly aligning the point of germ tube emergence toward or away from the chemotropic source or by redirecting tip growth accordingly. At this earliest developmental stage, chemoattraction was most pronounced toward self-signaling cues (6-PP, SnTa and CWTa), plant-derived signals (13(s)-HODE) and simple metabolites (glucose and glutamine). The preference for simple C- and N-sources is obvious as they are easily metabolized and thus promote rapid cell growth and development. The high sensitivity of *T. atroviride* germlings toward plant oxylipins correlates to recent reports showing that conidial germlings of *T. harzianum* are strongly attracted to 13(s)-HODE (Lombardi et al., 2018). This provides further evidence that chemotropic recruitment of mycoparasitic fungi to (stressed) plant roots may be part of a plant defense mechanism against fungal plant pathogens (Hermosa et al., 2013). The chemotropic self-preference of conidial germlings reflects the need for establishing an interconnected germling network by cell-cell fusion as key step of colony initiation (Read et al., 2009; Roca et al., 2005). Together, both processes promote colony development and plant symbiosis and thus the rapid establishment of a fungal network in the rhizosphere.

Although conidial germlings responded differentially to culture supernatants of “easy” (SnBc and SnRs) and “difficult” (SnFo and SnFg) prey fungi, the relative weakness of these responses compared to *Trichoderma* self-signaling and plant association suggests that the perception of potential prey fungi has no major relevance at this early developmental stage. Although the attachment of *T. harzianum* germlings to prey hyphae has been documented (Inglis et al., 1999), there is no evidence so far that this physical interaction results in localized prey killing. Consequently, the low responsiveness of conidial germlings toward compounds from prey fungi suggests that they are still not able to perform a mycoparasitic attack. This makes biological sense considering that: (1) their small size limits the surface area of physical interaction with the fungal prey and also their ability to induce local nutrient limitation or space competition, (2) they lack secondary metabolites and sufficient metabolic activity to significantly modify the surrounding environment through content secretion, and (3) there is no evidence for conidial germlings of *Trichoderma* species being able to kill prey hyphae through penetration and invasive growth. In contrast, all of these features have been widely observed in mature hyphae of mycoparasitic *Trichoderma* species (Benítez et al., 2004). The symbiotic association of conidial germlings to plants, including endophytic colonization of root tissue, on the other hand, is well documented (Chacón et al., 2007; Romão-Dumaresq et al., 2016; Zhang and Zhuang, 2020).

Interestingly, 6-PP has been related to inhibition of fungal plant pathogens (Serrano-Carreón et al., 2004; Rubio et al., 2009) and promotion of plant and root growth (Garnica-Vergara et al., 2016; Lee et al., 2016). The direct impact of 6-PP on *Trichoderma* morphology, however, has not been studied in detail. Studies so far have mostly focused on the metabolism and the physiological implications of 6-PP production (Serrano-Carreon et al., 1993; Rocha-Valadez et al., 2005). Hence, in terms of self-signaling, the role of 6-PP in cell fusion requires further evaluation. It was previously demonstrated that the ability to establish cell fusion is essential for efficient mycoparasitic activity of *T. atroviride*, using mutants lacking key components of the MAPK pheromone response pathway (Gruber and Zeilinger, 2014).

Notably, 6-PP is an oxygenated compound which was previously suggested to belong to the oxylipin family (Serrano-Carreon et al., 1993; Howe and Schilmiller, 2002; Zeilinger et al., 2016; Speckbacher et al., 2020a). This implies that independent of their origin, compounds of similar oxylipin-like structure may elicit strong chemotropic responses in *Trichoderma* – and probably other fungal species – and thus may constitute an interspecies signaling mechanism. In the context of mycoparasitism it is compelling to speculate that the plant oxylipin functions in recruiting the mycoparasite toward roots affected by fungal plant pathogens – or already as precautionary measure – while the fungal oxylipin (6-PP) induces morphological changes in *Trichoderma* required to launch a localized mycoparasitic attack. Our findings in the different developmental stages provide some support for this idea.

With the developmental transition from germ tube to hyphae, detection of prey fungi becomes relevant. This is most evident with the SnRs F30 fraction, where the chemotropic response of hyphae constituting the periphery of microcolonies toward prey-derived signals exceeded that of the other two fractions and the glucose control. Notably, the phenotype of this response did not result in obvious directional changes of tip growth of individual hyphae, but rather in an increase of the overall colony extension rate and hyphal density in the region closest to the chemoattractant source. This suggests that addition of nutrients available from prey-derived resources becomes biologically important for *T. atroviride*, even though it is not obligate under experimental conditions. In a nutrient poor natural habitat, however, this effect could be highly relevant, especially during the intermediate developmental stage of the microcolony which is still limited in size and cannot accumulate significant internal reserves. Therefore, besides searching for a potential prey in the vicinity, self-signaling compounds and plant oxylipins remain important chemotropic cues to advance the expansion of an interconnected symbiotic mycelium. Provision of nutrients from the plant host are likely to play an important role for the rapid transition to a fully developed mature colony.

The differentiation of morphological responses also points toward early changes of metabolic activity. SnTa and 13(s)-HODE, for instance, most notably promoted colony extension, whereas 6-PP primarily led to an increase in hyphal density. Similarly, SnRs F10 noticeably restricted colony extension, while SnRs F30 significantly increased hyphal density. The longer the exposure, the more pronounced these distinct features developed. The onset of mycoparasitic prey killing has already been associated with the induction of hyphal multipolarization (Lu et al., 2004). Hence, we speculate that the perception of prey-derived signals also triggers localized enlargement of available secretory and probably absorption surfaces to prepare the colony region closest to the signal source for imminent prey lysis and nutrient uptake, along with the required metabolic adaptations.

Full colony development generated mature hyphae with high sensitivity toward prey-derived signals. The SnRs F30 fraction induced a positive directional growth response that exceeded that of the glucose control and brought the colony periphery into close contact with the compound source. This marks the point at which fungal prey sensing overrides sensing of nutrients available in the growth medium. The fact that strong inductive cues can switch the morphological and metabolic program to an unnecessary behavior, because there are sufficient nutrients provided in the culture medium, is long known from (hemi-)cellulase induction in the related species *T. reesei* (Sternberg and Mandels, 1979; Kubicek et al., 2009; Lichius et al., 2014) and has been shown at the level of altered gene expression in T. *atroviride* (Atanasova et al., 2013). As mentioned before, in a nutrient poor soil environment it is essential for survival of the fungus to swiftly exploit a potential prey fungus as food source. The chemorepellent SnRs F10 fraction, on the other hand, restricted localized extension of the colony periphery, preventing its contact to the compound source. This is equally important in order to avoid investing valuable growth resources into nutritional “dead ends.” Together, these findings suggest that mature hyphae of the colony periphery are the primary sensors and signaling responders to prey-derived chemotropic cues, and consequently, are responsible for eliciting the mycoparasitic attack program only when appropriate. Initiating signals are likely to be passed to the sub-peripheral region, in which further morphogenetic and metabolic adaptations prepare subsequent prey lysis and nutrient uptake. However, in contrast to microcolonies, localized regions that drive colony edge expansion and others that increase secretion and absorption areas can no longer be discriminated at this mature developmental stage due to the high hyphal density.

The mass release of 6-PP at this stage would not only have an inhibitory antifungal action, but might work as a signal highly secreted in the regions of the colony in contact with the prey fungus, in order to promote hyphal growth and hyphal redirection as a mechanism of mycoparasitism. Additional secondary metabolites secreted by *T. atroviride* are likely involved during these events to support and refine the chemoattraction process and further mycoparasitism, indicated by consistently strong chemotropic responses to SnTa in all assays.

## Conclusion

In conclusion, each of the three assays described here evaluates different aspects of morphological responses to chemotropic compounds. Conidial germlings emerged as being ideal for chemotropic compound screening aimed at the rapid identification of strong chemoattractants and chemorepellents, as already reported by others (Turrà et al., 2015; Lombardi et al., 2018). High CI values while evaluating complex chemotropic compounds, such as SnBc and SnRs, directly correlated with the ability of *T. atroviride* to efficiently parasitize the corresponding prey species in standard confrontation assays, identifying them as “easy preys,” while low CI values correlated with reduced mycoparasitic activity identifying “difficult preys,” such as *F. oxysporum*. Backed up by these correlations, the germling format produced results within a few hours instead of days required in macrocolony-level experiments. Furthermore, liquid germling assays performed in a micro-well format allow automated high content screening and are therefore increasingly relevant for industrial applications. Microcolonies represent an intermediate state that allows studying how molecular and cellular features of chemotropism adapt until the fungal colony reaches full maturity. Therefore, microcolonies, together with expansion-limited mature colonies, are essential for investigating the effects of chemotropic compounds on mature hyphae and on particular cellular processes that occur only in the context of an interconnected and differentiating fungal colony. Consequently, all three approaches are best used in combination to gain comprehensive insights on chemotropic signaling throughout filamentous fungal development. Our study also indicates that the concentrations of prey-derived chemoattractants are presumably too low in the complex SnBc, SnRs, and SnFo mixtures to induce morphological changes visible at the macroscopic and low-magnification microscopic levels. Simple molecular fractionation allowed detection of such responses and may provide deeper insights. More sophisticated molecular fractionation, concentration and purification methods of culture supernatants from prey fungi has thus a high potential to identify novel compounds able to induce chemotropic responses in *T. atroviride* or in other mycoparasitic species.

## Data Availability Statement

The original contributions presented in the study are included in the article/Supplementary Material, further inquiries can be directed to the corresponding author.

## Author Contributions

DM-R, AL, and SZ conceived and directed this study and drafted the manuscript. DM-R performed the experiments. DT and AD contributed in the experimental design of the chemotropism assay with conidial germlings and hosted DM-R in their lab for 4 weeks. All authors read, revised, and approved the manuscript.

## Conflict of Interest

The authors declare that the research was conducted in the absence of any commercial or financial relationships that could be construed as a potential conflict of interest.
