# Supplementary material for: Chemotropism Assays for Plant Symbiosis and Mycoparasitism Related Compound Screening in Trichoderma atroviride
Source: Front Microbiol. 2020 Nov 27;11:601251. doi: 10.3389/fmicb.2020.601251 (PMC7729004; doi:10.3389/fmicb.2020.601251)
Supplement: Supplementary file 1 [file Data_Sheet_1.docx]

Chemotropism assays for plant symbiosis and mycoparasitism related compound screening in *Trichoderma atroviride*

**Dubraska Moreno-Ruiz^1,‡^, Alexander Lichius^1, ‡^, David Turrà^2,3^, Antonio Di Pietro^2^ and Susanne Zeillinger^1^**

^1^University of Innsbruck, Department of Microbiology, Innsbruck, Austria

^2^Universidad de Córdoba, Departamento de Genética, Córdoba, Spain

^3^Current address: University of Naples Federico II, Department of Agricultural Sciences, Portici, Italy

**Supplementary data**

**
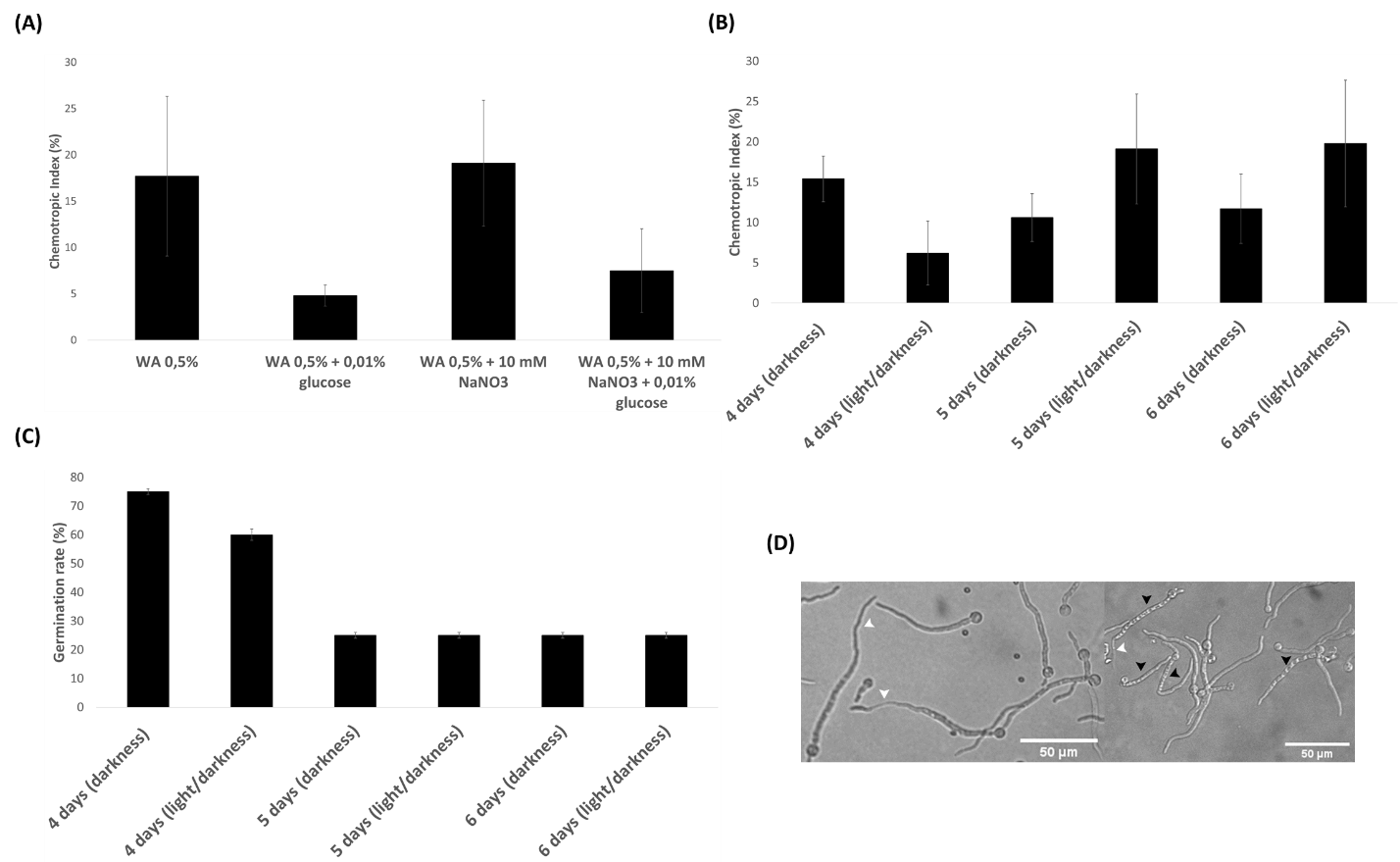
**

**Supplementary Figure S1.** **Chemotropism of *T. atroviride* is significantly affected by the age and pre-incubation settings of conidial germlings and by the culture medium composition. (A)** Glucose chemotropic index comparison between water agar and variations in provided glucose and NaNO_3_ content. The maximum CI obtained was close to 20 % when water agar was supplemented with NaNO_3_. **(B)** Glucose chemotropic index for varying germlings age and incubation conditions using water agar with 10 mM NaNO_3_. Five to six days old conidia under light/dark pre-incubation conditions were best suited for chemotropism assays. **(C)** Germination rate during chemotropism with glucose varying germlings age and light conditions, using water agar with 10 mM NaNO_3_. Germination rate was higher when four day-old conidia under dark pre-incubation conditions were used in the chemotropism assay, while five and six-day old conidia displayed a three-times reduced germination rate. **(D)** Germlings of *T. atroviride* displaying stress morphology in a chemotropism assay on M9 agar. Tips of germ tubes (white arrow) and high vacuole presence (black arrows) were the main morphological modifications observed during chemotropism assays with M9 agar. Medium carbon and nitrogen content, and pH values were modified to improve morphological and chemotropic index features.

**
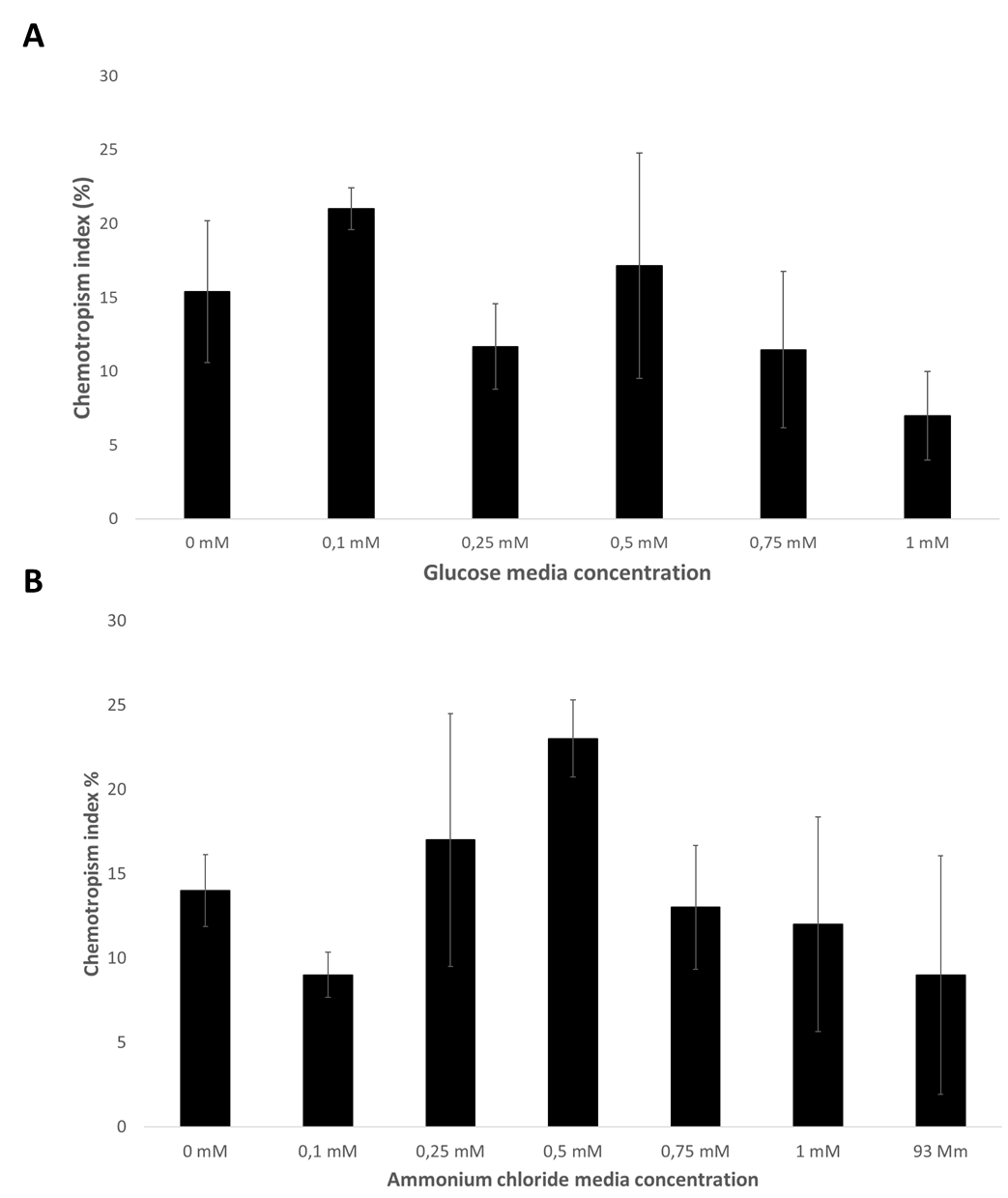
**

**Supplementary Figure S2.** **Chemotropism assay using *T. atroviride* germlings in the presence of varying glucose and nitrogen content in M9 media. (A)** Glucose chemotropic index comparison between M9 media with glucose content variations. (**B)** Glucose chemotropic index comparison between M9 media with ammonium chloride content variations.
